# Nudging oral habits; application of behavioral economics in oral health promotion: a critical review

**DOI:** 10.3389/fpubh.2023.1243246

**Published:** 2023-12-08

**Authors:** Ali Kazemian, Melika Hoseinzadeh, Seyed Ahmad Banihashem Rad, Anahid Jouya, Bahareh Tahani

**Affiliations:** ^1^Department of Community Oral Health, School of Dentistry, Mashhad University of Medical Sciences, Mashhad, Iran; ^2^Dental Research Center, Mashhad Dental School, Mashhad University of Medical Sciences, Mashhad, Iran; ^3^Department of Restorative, Preventive and Pediatric Dentistry, University of Bern, Bern, Switzerland; ^4^Graduate School for Health Sciences, University of Bern, Bern, Switzerland; ^5^Department of Oral Public Health, Dental Research Center, School of Dentistry, Dental Research Institute, Isfahan University of Medical Sciences, Isfahan, Iran

**Keywords:** oral health, dental care, choice architecture, nudge, behavioral economics

## Abstract

**Background:**

Oral health disorders significantly contribute to the global incidence of chronic diseases. Nudge interventions have demonstrated effectiveness in enhancing people’s decision-making and self-management capacities in a cost-efficient manner. As a result, these interventions could be valuable tools for fostering improved oral care habits. This critical review explores potential behavioral nudges applicable to promoting oral health.

**Methods:**

A thorough electronic literature search was conducted on Scopus, Embase, and PubMed databases for papers published post-2008. The search focused on empirical evidence concerning the direct and indirect application of Nudge theory in oral health enhancement. In addition, the investigation included the nudge intervention’s role in managing common non-communicable disease risk factors (tobacco, alcohol, and sugar) and their use in other health sectors.

**Results and conclusion:**

There is a dearth of studies on behavioral economics, particularly those involving reward and reminder techniques. However, various successful nudge interventions have been identified in other sectors that aim to improve health decisions. These include strategies encouraging healthier nutritional choices, tobacco and alcohol cessation, medication compliance, routine physical activity, and regular health check-ups. Such interventions can also have direct or indirect positive impacts on oral health. Implementing these interventions within an oral care framework could promote oral health due to similar underlying cognitive mechanisms. However, different types of nudge interventions have varying degrees of effectiveness. Furthermore, factors such as the method of delivery and the characteristics of the targeted population significantly influence the outcome of the intervention. Hence, it is imperative to conduct extensive studies in diverse socioeconomic settings to fully understand the potentials, limitations, and impacts of nudge interventions in promoting oral health.

## 1 Introduction

Behavioral economics is a new field of social study that uses the findings of psychology in economics. Two Nobel Prizes in Economics for Daniel Kahneman in 2002 and Richard Thaler in 2017 brought behavioral economics to particular academic attention in different disciplines. Thaler’s theory, known as Nudge Theory, deals with cheap and easy interventions that effectively change people’s behavior. Nudge theory focuses on Easy, Attractive, Social, and Timely interventions (EAST) to encourage desirable and healthy behaviors.

Although it seems logical that people would make the best health decisions, many continue to prioritize short-term pleasures despite being aware of the long-term negative effects on their health (1). Nudge theory acknowledges behavioral complexity and rejects the idea that humans would make optimum decisions when given the right information (2). Instead, nudges are used as interventions that are neither mandatory nor choice-restricting but design choices effectively and desirably.

There is promising evidence that nudges can be used to improve a wide range of health policy domains, including preventive healthcare. According to the World Health Organization (WHO), focusing on the most cost-effective and feasible interventions to prevent and control noncommunicable diseases in low-and middle-income countries could save close to 7 billion lives by 2030 (3). Noncommunicable diseases, such as type 2 diabetes, have been investigated as potential nudge intervention targets (4). Nudges have also been shown to have a positive impact on patient’s lifestyle choices, such as diet, medication adherence, and physical activity, as well as the use of tobacco and alcohol (5–7). Previous systematic reviews found that the majority of current nudge studies were conducted in nutritional sciences, which is critical for other health topics such as oral health (8–10).

Oral health diseases, i.e., dental caries, periodontal diseases, and oral cancer, are among significant contributors to the worldwide burden of chronic diseases (11). Poor oral health has a detrimental effect on one’s quality of life and may raise one’s chance of developing chronic diseases (12). For instance, prolonged discomfort from an infected tooth might impair food intake and nutrition. Moreover, evidence supports that bacteria associated with chronic periodontitis might be linked to diabetes and cardiovascular disease (13). Besides the importance of considering social and commercial determinants of oral health, there is abundant evidence for the significance of proper oral self-care, e.g., adequate and frequent tooth brushing and controlling sugar intake to prevent oral diseases. Healthy oral habits include eating healthily (8), brushing and flossing adequately and properly (14), and regular dental checkups (15), all of which depend on people’s self-management. Nudging, which targets better and healthier choices and adopting strategies to promote self-management, could be useful in improving oral care habits and decreasing the burden of oral disease. However, there is little existing literature about the nudge implications in oral health; therefore, this critical review aimed to synthesize behavioral nudges that can be used to directly or indirectly promote oral health. A Better understanding of behavioral Nudges might assist policymakers, clinicians, and researchers in developing and implementing useful nudge interventions to improve oral health.

## 2 Method and materials

The critical review protocol was registered at OSF Registries with registration code (https://doi.org/10.17605/OSF.IO/7FXCV) and is based on Daly and Carnwell’s framework (16) for the critical review, which includes determining the scope of the critical study, identifying and selecting relevant data sources, reviewing studies, and summarizing and categorizing the obtained evidence.

### 2.1 Scope of the review

This review endeavored to answer the following research question: “What are Nudge theory applications in developing healthy oral habits?”

Regarding the limited available evidence on the effective direct application of the Nudge theory in oral health promotion, papers in the other health sectors and oral disease risk factors (tobacco, alcohol, and sugar consumption) that were found potentially relevant were also included. The focused question was developed following the SPIDER tool (17):

***S*** (Sample): Health, Oral Health.

***P*** (Phenomenon) of ***I*** (Interest): All the articles that related to Nudge theory interventions.

***D*** (Design): not restricted.

***E*** (Evaluation): behavior change.

***R*** (Research type): not restricted.

### 2.2 Search strategy and terms

An electronic search was undertaken using Scopus, Embase, and PubMed databases for literature published after 2008. Authors extracted text words from relevant papers’ titles, abstracts, and index keywords to identify the articles. A search string was created using the keywords and synonyms in conjunction with the Boolean operators “AND” and “OR.” Only papers published in English were considered. All age groups were included. Search terms included combinations, plurals, and various conjugations of the words relating to identified nudge strategies. we set our search limit at 2008, since the conceptualization of the nudge theory, first introduced to a wide audience by Thaler and Sunstein in their 2008 book, Nudge: Improving decisions about health, wealth, and happiness (18).

(nudge[Title/Abstract] OR nudges[Title/Abstract] OR nudging[Title/Abstract] OR “choice architecture”[Title/Abstract] OR (“behavioral economics”[Title/Abstract] OR “behavioural economics”[Title/Abstract] OR “behavioral model”[Title/Abstract] OR “behavioural model”[Title/Abstract] OR “behavioral control”[Title/Abstract] OR “behavioural control”[Title/Abstract] OR “behavior control”[Title/Abstract]) AND “health promotion”[MeSH Terms] OR health promotion[Text Word] OR “oral health”[MeSH Terms] OR oral health[Text Word] OR “health”[MeSH Terms] OR Health[Text Word]).

### 2.3 Reviewing the studies

After eliminating duplicates, the authors conducted a comprehensive review and provided a summary of the selected literature. The primary focus was on oral health promotion and behavior changes across various populations. The decision to include specific resources that demonstrated the effectiveness of the Nudge theory in sectors beyond oral health promotion was reached through consensus among the authors during focus group discussions.

**Figure 1:**
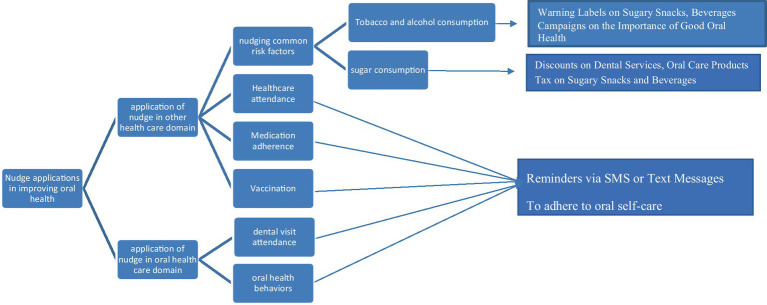
Concept map showing the resources selection procedure and key applications of Nudge theory in improving oral health.

To select these resources, two main approaches were taken into consideration. Firstly, the common risk factor approach was employed, where articles highlighting the successful application of the Nudge theory in modifying common behavioral risk factors associated with non-communicable diseases such as tobacco, sugar, and alcohol were collected. These risk factors have been established to impact oral health concurrently. Additionally, other resources that indicated the efficacy of the Nudge theory in modifying or promoting self-care behaviors with shared cognitive mechanisms, such as regular physical activity, attending physician visits, and medication adherence, were also included (Figure 1).

## 3 Results and discussion

### 3.1 Studies on nudging oral habits

Despite the growing popularity of nudge theory in various fields, including economics, public policy, and healthcare, there is a noticeable lack of research on its applications in oral health and dentistry. While nudges have been shown to be effective in promoting healthy behaviors and improving patient outcomes in other healthcare settings, such as smoking cessation and medication adherence, their potential impact on oral health behaviors remains largely unexplored. In this part of the review, we aim to report the limited papers on nudge theory in oral health and dentistry.

One of the most relevant papers on nudging in dental settings is a perspective article by Scarbecz (19). The author discussed how dental team members could use behavioral economics principles to improve patients’ oral health and lead patients to make healthy choices. Patients’ choices will be influenced by the way health information is provided to them, dental team members should use the best ways to present information to patients to improve their welfare and preserve their autonomy. It has been argued that the decision-making process for dental patients is usually complex and difficult due to a number of economic, medical, and psychological factors. As dental patients often do not receive immediate feedback on their treatment outcomes, nudges might be helpful. Scarbecz discussed common decision-making biases in dental settings, such as anchoring, availability heuristics, frequency-based judgments, optimism, status quo bias, and conformity. Using the “choice architecture” concept, he proposed practical strategies. For example, dental teams could offer incentives such as discounts for positive oral health behaviors during recall appointments, using decision diagrams that outline treatment options and their implications, and implement feedback mechanisms to counter the lack of immediate rewards for behaviors such as flossing. Patients might make better decisions about their oral health by using these approaches. However, it was a perspective study and not all of the interventions proposed by the author were tested or applied clinically.

In a recent study, Shariati et al. (20) investigated the self-reported oral health of a random cluster of residents in Mashhad in relation to their estimation of the oral health of the majority of people in Mashhad. They found a positive correlation between the self-and others’ oral health levels and decayed and missing teeth (DMT). Their findings indicated that people might be “nudgeable” for behavioral change by social norm interventions.

In a commentary and discussion paper, Wang and Wang (21) attempted to elucidate how behavioral economics can help clinicians analyze patients’ fear of COVID-19 and assist them in making an informed treatment choice. Based on behavioral economics principles, it is asserted that clinicians are able to determine how fear of COVID-19 infection can influence patients’ decisions and, consequently, oral health outcomes. The risk compensation bias suggests that some patients may overestimate the risks of COVID-19 and underestimate the advantages of receiving care. While the dangers of the pandemic are unquestionably grave, they may be exaggerated due to availability bias or the tendency of humans to place a greater cognitive weight on information that is more readily accessible. Therefore, clinicians have to reduce the risk of COVID-19 exposure and, through effective communication, clarify the disadvantages of delaying treatment. They can frame their explanation as a gain or a loss. Additionally, it may be beneficial to clarify what other patients in comparable situations have chosen to do and leverage social norms.

In a field study conducted by the Social Policy Institute at Washington University in St. Louis (22, 23), the effect of two nudges intended to encourage parents of infants to participate in Teeth Brushing Meetings (TBM) was evaluated among 2,050 children in 41 daycare centers. Parents were reminded to take care for their children’s oral health in the weeks preceding the TBM. To serve as a daily reminder in the 2 weeks preceding the oral health workshop, a collaborative poster board was placed at the entrance of the nursery classrooms, asking parents to place stickers in two columns labeled “We brushed our child’s teeth” and “We did not brush our child’s teeth.” The second nudge was intended to remind parents of the possible benefits (vs. losses) of good (vs. poor) oral hygiene routines using differing wording in their invitation letters; the control group was given a neutral invitation letter to learn about caring for their child’s oral health. The second group (nudge) received a “Negative accountability letter,” which reminded them that their child’s poor oral health is their responsibility. The letter included a graphic depicting the consequences of poor oral hygiene. The invitation letter of the third group, or “Positive accountability letter” group, illustrated a beaming child with a healthy mouth and emphasized that children’s oral hygiene results due to parents meeting their responsibility. According to the findings of the study, the interventions successfully increased parental participation in the TBM. However, there was not a substantial distinction between the three groups.

In another experiment (24) in Early Head Start (EHS) sites in the Los Angeles, California area, the BEECON (Behavioural Economics for Oral Health Innovation) pilot trial was carried out during 8 study weeks based on the concepts of nudging behavior with appropriate incentives. This project was to motivate low-income parents of children aged 1–3 to brush their children’s teeth frequently and attend scheduled dental visits by evaluating and contrasting the concept of a fixed monetary incentive package, a combined lottery incentive package (to capitalize on the propensity for individuals to be more inspired by immediate rather than delayed gratification), and a waiting list (delayed incentive) control. During the study period, participants were provided with Bluetooth-enabled powered toothbrushes that synchronized data to a mobile app in order to monitor toothbrushing compliance. In the fixed incentive group, participants received $5 per week if they met an inferior performance threshold (7 episodes, daily, 1 min) and $10 per week if they met a higher performance threshold (14 episodes, twice daily, 1 min). The lottery monetary incentive group obtained a weekly SMS regarding their entry into a lottery drawing. The likelihood of success relied on the participant’s level of accomplishment. After 8 weeks of study, participants in the control group earned the same amount of money as those in the fixed incentive group. All participants received messages regarding their brushing performance and reminders to integrate their toothbrushing data with the app. In the lottery incentive group, the mean number of weekly grooming episodes over 8 weeks was 6, compared to 4.1 in the fixed incentive group and 3.9 in the control group. The lottery group reported brushing their teeth 53% more frequently than the control group and 47% more frequently than the fixed group. It was determined that an integrated (two-tiered) lottery incentive program is a viable method for encouraging good dental hygiene in young children.

In another commentary article, Wang et al. (22) sought to demonstrate how behavioral economics might be used to reduce missed dental appointments. They first outlined the basic issues that may be caused by these missed appointments, such as the exacerbation of dental cavities and the complication of dental treatments, as well as the strains on the patient-provider relationship caused by the erosion of trust. Again, Wang et al. (22) addressed salience bias, present bias, planning fallacy, and risk compensation bias as the related cognitive-behavioral obstacles and then suggested a strategy to illustrate how these insights could be combined to reduce missed appointments. Then, they offered suggestions based on the principles of behavioral economics, such as substituting the term “dental cleanings” with “oral health examination” when communicating with patients in person or via reminder messages, and mentioning the advantages of an oral health examination. The reminder text may briefly summarize the effects of oral diseases such as periodontitis and oral cancer. Additionally, they recommended integrating pre-commitment devices into the automated text reminders. For instance, a reminder can be sent to the patient 1 week prior to the appointment, necessitating active affirmation.

One useful strategy in addressing patient reluctance or a “wait and see” attitude is to emphasize the potential financial implications of delaying necessary treatment. Healthcare providers can effectively raise patient awareness of the importance of timely treatment by highlighting the long-term consequences and expenses associated with treatment delay. This approach enables patients to comprehend that the immediate financial investment in treatment is typically outweighed by the physical distress and financial strain that can arise from the progression of disease. Consequently, patients are more inclined to undertake appropriate measures promptly rather than postponing action (19).

In total, what we have in accordance with the implication of behavioral economics in dental care are mostly hypothetical suggestions based on the cognitive bias of humankind and the proposed solutions that might arise from the principles of behavioral economics. There are also some limited empirical studies piloting the use of these principles, especially rewarding and reminding, to steer the oral hygiene behaviors (such as toothbrushing or dental visit attendance) in parents of children. However, behavioral economics is not limited to being relevant only to oral hygiene behaviors, and the other relevant fields need to be tested through clinical or field trials.

### 3.2 Studies on nudging common risk factors

#### 3.2.1 Nutritional choices and sugar consumption

Nudge theory has implications for improving oral health outcomes by influencing sugar, tobacco, and alcohol consumption. Most nudge interventions have been studied in nutritional sciences; therefore, we used the previous reviews in this field. Studies have reported a moderate effect of nudge interventions on food choices and nutritional decisions (25). Recent research by Mertens et al. (26) on the influence of nudging across behavioral domains indicated that choice architecture interventions encourage behavior change with a small to moderate effect size. The effect magnitude of food choice nudges, according to the authors, was 2.5 times greater than that of other behavioral domains (26). According to a meta-analysis by Arno et al. (9), nudge interventions, on average, increases healthier dietary or nutritional decisions by 15.3%, measured by changes in healthy choices frequency or overall caloric intake.

The main nudge interventions for diet were categorized in a systematic review and meta-synthesis as (1) repositioning and replacing food items, (2) food items presentations in the form of amounts and servings, (3) using posters, calorific labels, stickers, and signs to promote healthy food choice, (4) using reminders in forms of text messages, emails, and online lessons, and applications to notify individuals about nutrition and healthy eating, (5) financial incentives, (6) affecting senses (sight, smell, and taste) to influence lunch choice or healthy food selection, and (7) cognitive loading, where cognitive resources for making decisions are restricted (27). A meta-analysis by Vargas-Alvarez et al. (28) revealed that specific portion control tools have small size effects and may be effective instruments for inclusion as part of weight loss interventions.

Managing diet and nutrition is one of the most important aspects of maintaining health in patients with noncommunicable diseases. In a systematic review, Kwan et al. (29) investigated the influence of nudge interventions on the diet of diabetic adults. They found that nudge interventions’ delivery mode is influential in changing patients’ behavior. Using social modeling delivered through group meeting sessions effectively modified the patient’s diet, physical activity level, self-efficacy, and HbA1c control. Whereas digital devices alert to reminded patients to eat less were ineffective.

The population’s socioeconomic status appears to influence the effectiveness of nutritional nudge strategies. Schüz et al. (30), systematically reviewed the equity effects of nutritional nudging strategies in individuals from different socioeconomic backgrounds. According to most of the equity comparisons in the literature, cognitive nudges (i.e., nudges that encourage information presentation about the food) worked similarly in more and less disadvantaged populations; however, in some studies, these kinds of nudges favored less economically disadvantaged people. In addition, they discovered that certain behavioral nudges (altering the accessibility or convenience of food options) favored disadvantaged individuals. In consistent with previous studies, Harbers et al. (25) declared that there is evidence that nudges were more effective in low socioeconomic status groups, but studies on these populations are scarce.

Regarding sugar consumption, Venema et al. (31) found that decreasing tea spoon size reduced sugar intake on average by 27% among participants. However, the nudge effect was less pronounced when people had a strong habit of adding sugar to tea. Villinger et al. (32) reported that modifying the sugar shakers’ design to release a smaller amount of sugar in each pour reduced added sugar by 20% over 4 weeks. In a randomized clinical trial, authors have found that sugar-sweetened-beverage consumption and healthier drink choice can be nudged by Instagram image priming (33).

In summary, nudge interventions have demonstrated the potential in swaying nutritional choices, thereby directly influencing oral health through the reduction of high-calorie and sugary products. The efficacy of these interventions, however, is not uniform and hinges on several factors such as the method of delivery and the socioeconomic status of the target audience. Various strategies, including food repositioning, reminders, and alterations in food presentation, have shown their effectiveness in this context.

#### 3.2.2 Tobacco and alcohol consumption

The nudge theory can promote healthier behaviors related to tobacco and alcohol cessation. Nudge interventions can be designed to provide support for individuals who want to quit tobacco or alcohol by offering reminders, incentives, or access to resources such as quit lines or counseling services. These interventions could help individuals overcome barriers to quitting and increase their motivation to adopt healthier behaviors, leading to improved oral health outcomes and overall well-being.

Research has shown that graphic warning labels on cigarette packs can nudge people toward quitting smoking. Nurchis et al. (34) discovered that enhancing the salience of information or incentives emerged as the most widely utilized nudge intervention, demonstrating a higher success rate when compared to other nudge strategies. The proposed underlying mechanism suggests that these interventions elicit negative emotional stimuli, including fear, worry, and disgust. Similarly, warning labels on alcohol bottles could remind people of the risks of excessive alcohol consumption, and raise their awareness. The scientific literature highlighted the larger effectiveness of image-based warning cues in avoiding dangerous activities (35). Fakir and Bharati (36) examined the efficacy of two behavioral strategies to reduce tobacco use in an ultra-poor rural area of Bangladesh, where traditional approaches such as taxes are impractical. The first strategy required participants to record their daily use of tobacco costs. The second strategy consisted of placing two graphic banners with warnings about the adverse impacts of tobacco use on tobacco users and their offspring. While both strategies decreased household tobacco consumption expenses, male participants who recorded their expenditures opted for inexpensive smokeless tobacco. Males who are married to non-smokers have a greater decrease in their tobacco consumption. An exploratory analysis showed that risk-averse males who invested a greater proportion of their income on tobacco replied better to the logbook strategy. Male patients with children younger than five and a higher level of education reacted more effectively to the poster strategy (36).

Clarke et al. (37) highlighted the effectiveness of Health warning labels (HWLs) on products containing tobacco and alcoholic beverages to decrease smoking and drinking. Three hundred and ninety-nine adults over the age of 18 who purchased beer or wine weekly for consumption at home make up the sample. Participants were randomly assigned to one of three groups based on the HWL displayed on the packaging of alcoholic beverages: (a) image-and-text HWL; (b) text-only HWL; and (c) no HWL. They found no obvious evidence of a difference between the HWL groups and the control group in terms of the quantity of alcoholic drinks selected. Substantial greater negative emotional arousal and lower acceptance were observed in the image-and-text HWL group relative to the text-only HWL group.

People are often influenced by what others around them are doing. Nudging people toward healthier behaviors can be achieved by highlighting the social norm of healthy behavior. For example, campaigns could potentially showcase how many people have quit smoking or reduced their alcohol intake. Highlighting social norms of healthier behaviors, such as emphasizing that most people do not smoke or only drink in moderation, can nudge individuals toward aligning their behavior with the norm (38).

One way to nudge people toward healthier choices is to change the default option. For example, making non-smoking the default option in public spaces or making low-alcohol beverages the default option in bars and restaurants can encourage people to make healthier choices without restricting their freedom. Nudge theory can be used to change the default option, such as making non-smoking or non-drinking the default option in certain situations. Hempel-Bruder et al. (39) evaluated the effectiveness of educating General Practitioners (GPs) to provide treatment as the default option with current tobacco users seen in primary care using an encounter decision aid. The use of default options and an electronic decision aid are low-cost, readily disseminable interventions. They hypothesized that general practitioners who provide smoking cessation treatment as the default option using an encounter decision aid will boost the percentage of patients who cease smoking (39).

Increasing taxes on tobacco and alcohol products can nudge people toward reducing their consumption. Studies have shown that higher prices could deter people from purchasing these products. Providing incentives for healthy behaviors could also nudge people toward healthier choices. For example, discounts on healthy food and beverage options or rewards for not smoking or drinking can encourage people to make healthier choices. Nudge theory can be used to provide incentives for behavior change. For example, a workplace could incentivize employees who quit smoking or reduce their alcohol consumption. Cho et al. (40) discovered that the cost of cigarettes had become the most frequently cited reason for quitting or cutting back on smoking, particularly for those living in low socioeconomic areas, consuming more cigarettes daily, drinking alcohol, and experiencing high/very high emotional distress. Since 2013, a change in the primary federal tobacco control strategy implemented in Australia, from mass-media campaigns to tobacco tax rises, has likely resulted in cost, rather than health, being cited as the main driver for altering smoking behavior (40).

Nudge theory can be used to provide information about the harms of tobacco and alcohol consumption in a way that is easily accessible and understandable. For example, putting up posters or signs in public places such as bars or restaurants that highlight the risks of smoking or drinking excessively. Jenssen et al. (41) conducted a trial in nine clinical sites within the Cancer Control Implementation Lab of the Implementation Science Centre to assess the effect of behavioral economic implementation strategies provided via embedded messages (or “nudges”) encouraging patient involvement with the Tobacco Use Treatment Service. Nudges were electronic medical record (EMR)-based messages sent to patients, clinicians, or both, intending to counter specific patient and clinician biases that reduce treatment engagement (41). Drake et al. (42) proposed a Clinical Decision Support (CDS) intervention to encourage clinicians to use the CDS instrument in order to increase tobacco cessation among tobacco users. Using user-centered design principles and the CDS Five Rights, they created a CDS tool that dynamically inserts useful data about current tobacco users into the Assessment and Plan section of clinicians’ notes. They evaluated the efficacy of the CDS tool on time to tobacco cessation among patients at four primary care practices in the Denver metropolitan area (42).

Providing individuals with timely feedback on their tobacco or alcohol consumption, such as through apps, can nudge them toward making healthier choices and monitoring their behavior. Bhatt et al. (43) emphasized the necessity of understanding and valuing the dynamics of social and cultural variables in order to develop an effective de-addiction strategy. The patient was provided with disease-specific pamphlets and SMS (short text messages) in their native language were provided with basic tips for handling his tobacco cravings (such as not purchasing tobacco pouches on his own and not requesting anyone else to do so). The patients received a counseling session to heighten their awareness of tobacco use. Participants were asked to watch videos about how their tobacco use contributed to diseases and enhanced risk of complications.

Drawing from the success of nudge interventions in tobacco and alcohol cessation efforts, similar strategies could be applied to promote oral health. Just as graphic warning labels on cigarette packs effectively nudge people toward quitting smoking, labels emphasizing the risk of oral cancers may heighten public awareness. One way to do this is by implementing graphic warning labels on oral health-related products, like sugary snacks or beverages, bringing the risks of poor oral health to the fore.

It’s also beneficial to emphasize social norms surrounding oral hygiene. Launching campaigns that underline the importance of maintaining good oral health can significantly improve public consciousness in this regard. Providing incentives can further motivate individuals to adhere to proper oral health behaviors. This could take the form of offering discounts on dental services or oral care products to those who comply.

Moreover, another effective strategy could be implementing a tax on sugary snacks and beverages. This financial deterrent might discourage excessive consumption, ultimately contributing to better oral health outcomes.

### 3.3 Modeling nudge interventions from other health-related conditions

The evidence of implementing nudges in promoting oral health is scarce. However, the components of oral health behavior are similar to other health-related behaviors among healthy populations and patients with chronic diseases. Based on the proposed health behavior taxonomies (44, 45), similar psychological factors or goal structures may underlie similar behaviors. Consequently, successful behavioral and nudging interventions for some types of behaviors might apply to optimizing others. In addition, targeting multiple behaviors in intervention programs is an effective method for maximizing efficiency and cost-effectiveness. Therefore, we investigated studies on other health promotion actions and hypothetically correlated them with oral health behaviors, including daily oral hygiene adherence, receiving oral disease preventive care, attending dental checkup appointments, and obtaining dental care insurance.

#### 3.3.1 Medication adherence/daily oral hygiene adherence

According to the WHO, adherence is the degree to which a person’s behavior corresponds with the agreed-upon recommendations of a healthcare provider (46). In this regard, daily oral hygiene adherence might be analogous to medication adherence because both should be repeated regularly to improve health. Approximately one-fourth of patients do not adhere to their prescribed medication regimens or medical advice, which increases morbidity, mortality, and healthcare costs (47). Numerous studies have been conducted to determine the effect of nudge interventions on medication adherence. Reminders via SMS or text messages are the most prevalent nudge strategies (48). Möllenkamp et al. (47) conducted a systematic review to determine nudges’ efficacy in enhancing self-management of drug consumption among patients with chronic diseases. Interventions such as medication reminders, social support, and feedback nudges significantly enhanced medication adherence in patients with heart disease. Using reminders also substantially improved asthma and stroke medication adherence. In another systematic review, Kwan et al. (29) investigated the nudge interventions’ influence on diabetes management in adults. Text messages/email reminders and a pedometer/device were found to have a significant impact on medication adherence. In contrast, Luong et al. (49) reported in a randomized clinical trial that reminder text messages increased medication refills in patients with cardiovascular diseases and a 7-day refill interval, but the effect was insignificant. In another randomized controlled trial, Horne et al. (50) found that personalized nudges using machine learning of subjects’ characteristics derived from psychographic assessment, demographics, social determinants, and the Intermountain Mortality Risk Score (IMRS) significantly improved patients’ statin adherence after 12 months follow-up.

Rumi et al. (51) examined the influence of using an inhaler with a Turbo+ device on asthma patients’ inhaler usage management. This device transmits medication usage information to a smartphone application, sends reminders and motivational prompt messages, and visualizes medication usage. The device substantially improved patients’ inhaler usage within 90 days; however, because the study lacked a control group, it cannot be ruled out that improvements in health behavior may have been attributed to standard care. Ding et al. (48) evaluated the influence of applying the theory of planned behavior and the nudge strategies (salience nudge, social nudge, and feedback) on taking anticoagulant therapy in a 6-month follow-up. The authors observed that patients’ medication adherence decreased in both groups; however, providing messages in WeChat groups, encouraging patients to share their medication usage experiences, and praising participants who gained high scores significantly improved medication adherence at the 3-and 6-month follow-ups. However, the described studies suggest that nudge interventions, mainly reminders, have led to short-term improvements in medication adherence. The nudge intervention delivery mode and the patient characteristics may impact the efficacy of interventions (29).

The barriers to regular oral care vary according to age and socioeconomic status. The most common barriers, however, are a lack of knowledge, time, a negative attitude, insufficient toothbrushing resources, and forgetfulness (52, 53). In addition to educational interventions, nudge interventions such as smartphone reminders, gamification, social nudges within the family or among peers, and prompts may also improve compliance with oral hygiene behaviors or regular dental visits. Moreover, encouraging individuals who adhere to oral care behaviors in social network groups, for example, for students, might also improve oral care behavior adherence. However, provided studies regarding medication adherence often reported the short-term efficacy of the interventions. Since oral health care is a lifetime activity, nudges that influence daily activities might be more relevant to the field of oral healthcare.

#### 3.3.2 Physical activity/daily oral hygiene adherence

Inactivity and excessive sedentary behavior increase the risk of developing noncommunicable diseases and can diminish a person’s lifespan (54). However, many adults and adolescents do not meet the recommended amounts of physical exercise (55). Physical activity is also commonly addressed for nudge intervention in chronic disease management. Möllenkamp et al. (47) discovered that nudge interventions such as reminders, planning prompts, feedback, behavioral contracts, and salience nudges successfully enhanced objective and self-reported physical activity in patients suffering from various chronic conditions. Similarly, Kwan et al. (29) also found that gamification and reminders had a substantial favorable result in diabetes management. Similarly, a recent meta-analysis found that gamified smartphone apps may boost physical activity in healthy individuals (55).

Systematic reviews of nudge interventions in healthy people found that prompt interventions primarily promoted stair use (54, 56, 57); however, stair use decreased after the interventions were removed, and many programs failed to show long-term positive impacts. Longer-duration interventions successfully maintained the habit when the intervention was removed. Individuals may acclimate to nudge intervention over time and no longer perceive it. According to Li et al. (58), wearable activity trackers improve exercise behavior but are inefficient at changing habitual behavior, such as light physical or sedentary behavior. Furthermore, participant characteristics and intervention elements were linked to efficiency.

Teeth brushing and exercise are among daily routines for health maintenance (44). Although the approach and effects of nudge interventions for increasing individuals’ physical activity vary, applying behavioral nudges, such as reminders, gamification, and prompts, may also enhance dental hygiene practice. However, nudge interventions aimed at encouraging exercise habits in healthy individuals appear to be effective only for a short term, as people tend to adapt to them quickly. Therefore, when designing nudge interventions to promote oral health behaviors, it may be necessary to not only extend the duration of these initiatives but also to offer a variety of nudges to prevent individuals from growing accustomed to them.

#### 3.3.3 Vaccination/receiving oral disease preventive care

Several meta-analyses have found that patient reminders, such as phone calls, SMS, postcards, mail, or a mix of these methods, improve children, adolescents, and adult immunization against numerous diseases (59–63). Eze et al. (60) discovered that these reminders are significantly more effective than in upper-middle-income countries in increasing childhood immunization coverage and that sending more than two SMS reminders improves the timely receipt of childhood vaccines than sending one or two SMS reminders. In contrast to the beneficial effect of reminder nudges, Levine et al. (64) discovered that customized voice call reminders led to 10.5% higher coverage of on-time childhood vaccination while advising vaccination opportunities by a community health volunteer and providing a small incentive led to 49.5% increase in vaccination in rural Northern Ghana. In circumstances where network availability and phone access are limited, the impact of nudge vaccination via voice calls may be limited. In contrast, according to the Oyo-Ita et al. (65) meta-analysis, financial incentives did not affect childhood immunization coverage in low-and middle-income countries. However, they discovered that health education at village meetings, home, and facility-based health education, and revised vaccination reminder cards might boost total vaccine coverage.

Jacobson et al. (66) discovered that, whereas public health messaging and financial incentives boosted COVID-19 vaccination intentions, they did not raise immunization rates among vaccine-hesitant people. The outcomes of the Sasaki et al. study (67) emphasized the importance of tailoring social norm nudges to the purpose and target audience.

A significant portion of the direct costs of providing dental health care is used to treat highly preventable diseases in children and adolescents, which is burdensome for patients, governments, and insurance providers. In moderate-and high-risk individuals, applying fluoride varnish or resin-based fissure sealants of permanent teeth can prevent occlusal caries (68). Due to the common preventative nature of vaccines and dental professional preventive treatments and the limited number of interventions required, the nudge interventions that have effectively increased vaccination rates and timeliness may also encourage parents to receive dental preventive treatments. Personalized reminders and incentives, in addition to parental education and organizational infrastructures, may improve parental attendance at the dental office.

#### 3.3.4 Healthcare attendance/attending dental checkup appointments

Regular dental visits allow for the early detection of oral diseases and prompt, cost-effective treatment of dental problems (69). Appointment non-attendance in the primary healthcare system is costly, reduces access to limited resources, and is notably prevalent among vulnerable individuals. Patients’ perceptions that regular dental treatment is unnecessary or unusual, accessibility, participant characteristics (socioeconomic situation and history of drug, tobacco, and alcohol use), waiting time in the virtual queue, inability to get time off from work/school, and forgetfulness are all factors that influence non-attendance and no-show ups in dental offices (70–73). As a result, identifying the obstacles to attending dental appointments and carefully analyzing the “nudgeable” barriers identified in similar studies on healthcare attendance may improve populations’ oral health.

Möllenkamp et al. (47) investigated the effects of nudge interventions on patients with chronic disease attendance to physicians. They discovered weak to moderate evidence that small financial incentives, reminders, and planning prompts have a favorable effect. Huf et al. (74) evaluated the influence of text messages with varying content on cervical screening attendance. They discovered that SMS messages from primary care physicians dramatically increased people’s attendance. Furthermore, based on these findings, the National Health Service Cervical Screening Programme launched a London-wide screening campaign using text messages, resulting in a 4.8% increase in attendance in 6 months. In South Africa, Friedman et al. (75) showed that providing small incentives and massage reframe boosted attendance at counseling sessions for Voluntary Medical Male Circumcision as a free preventive treatment to reduce HIV infection.

Regarding no-show-ups, Boksmati et al.’s meta-analysis (76) showed that SMS appointment reminder within 48 h is an effective and operative method in decreasing appointment no-show-ups in a healthcare setting. In contrast to these findings, Ruggeri et al. (6) discovered that reminders did not affect disadvantaged people’s attendance. They looked at 53,149 visits and discovered whether patients were assigned to established physicians and appointment lead time was among the strongest predictors of no-show rates. According to the authors, underserved groups face numerous healthcare challenges. As a result, evaluating obstacles and planning treatments that target people in need is critical for the effectiveness of healthcare programs, including dental appointments and screening.

#### 3.3.5 Health insurance and retirement savings/dental care insurance

Private health insurance is usually a critical resource in covering dental care costs. Behavioral interventions might encourage people to choose their insurance plans more efficiently, along with the need for policymakers and insurance companies to provide more convenient dental insurance packages. Furthermore, insurance influences health-seeking behavior and oral health, particularly among vulnerable groups (77, 78). Many people often do not acquire health insurance, struggle to find appropriate coverage, or transfer plans despite changing needs. Based on the available evidence, Krishnan et al. (79) highlighted the behavioral traits and interventions that might steer consumer decision-making in health insurance market purchasing. The behavioral interventions were categorized as decision information-based, decision structure-based, or decision assistance-based. Successful nudges included framing, simplicity, social norms, defaults, sorting, callouts, labeling, reminders, and personalized information. They can motivate consumers to get dental insurance and improve the quality of their options. However, as the authors pointed out, there was a shortage of data from low-income and developing nations, and thus, the findings may not directly apply to these countries. In another study, Marzilli Ericson (80) found that nudging through letters or emails increased health insurance purchases in Colorado, but personalized nudges did not result in plan switching.

The prevalence of chronic non-communicable diseases, including oral disease, and demand for their treatment rise as people live longer. As a result, numerous nudge treatments to encourage early retirement savings have been examined. Even experts believe that financial literacy and awareness of the significance of saving are insufficient to motivate people to act. García and Vila (81) discovered that the default choice greatly enhances long-term voluntary savings of financially literate pension contribution system participants. Beshears et al. (82) discovered that framing the future time point around a fresh start date (e.g., the recipient’s birthday) enhanced the participants’ likelihood of contributing to a saving plan in a large-scale randomized field experiment on university employees. Dur et al. (83) studied the influence of social norm nudges on household buffer savings in a large-scale randomized field experiment at a retail bank. They discovered that while the norm nudge boosted individuals’ saving intentions, it did not enhance their savings. This study emphasized the methodological aspect of conducting nudge interventions, suggesting that using data other than final decisions may lead to researchers wrongly claiming that the intervention had an effect. Therefore, given that individuals might be receptive to nudges concerning oral health behaviors (20), it is vital to explore the real impact of norm nudges on concrete clinical measures such as plaque index and the long-term status of decayed, missing, and filled teeth (DMFT).

Some criticisms of nudge strategies assert that the philosophy of nudging contrasts with holistic, people-centered health-promoting interventions that incorporate the social and moral aspects of the setting approach (84). Others argue that with nudging, behavior and education are detached; education and contexts are prioritized over behavior (27). Moreover, studies show great heterogenicity that arises from study design, the method of measuring the efficacy of the interventions, sample group characteristics, and publication bias. Furthermore, most studies are conducted in high-income Global North nations, particularly in the United States. Therefore, Szaszi et al. (85) have argued that scholars must investigate when and where some nudges have huge positive effects. Since then, there has been “No reason to expect large and consistent effects of nudge interventions.”

Some authors support nudge interventions despite the critiques and the need for further investigations and high-quality studies in different cultures and settings. For instance, Benartzi et al. (86) discovered, for instance, that the ratios of effect to cost for nudge interventions frequently compare favorably with conventional policy tools, such as tax incentives and other financial inducements. Moreover, previous research has shown that using choice architecture to complement more traditional intervention approaches can enhance the impact of economic interventions such as taxes or financial incentives (87, 88). Mertens et al. (26) also stated that nudge interventions facilitate behavior change across various behavioral domains, population segments, and geographical locations. Therefore, we argue that nudge interventions can be useful for oral health promotion. Oral health is a multi-component phenomenon. We have described multiple nudge interventions that are directly conducted on oral health promotion or indirectly can affect oral health by influencing diet and tobacco consumption. We also explained interventions in other fields analogous to oral health components. Therefore, using different kinds of nudge interventions simultaneously by studying cultural elements and as a complement to other health promotion techniques might lead to significant outcomes (Table 1).

**Table 1:** Summary of the results and included studies in the review.

Health/oral health care	Domains	Sub-domains	Key research papers	Nudging related interventions	Implications for oral health
Implication of Nudge theory in other health domains with potentials for oral health related outcomes	Nudging common risk factors	Nutritional choices	Arno et al. (9), Harbers et al. (25), Mertens et al. (26), Ledderer et al. (27), Vargas-Alvarez et al. (28), Kwan et al. (29), Schüz et al. (30), Venema et al. (31), Villinger et al. (32), Kay (33)	Repositioning and replacing food items, food items presentations in the form of amounts and servings, using signs to promote healthy food choices, using reminders to notify individuals about nutrition and healthy eating, financial incentives, affecting senses to influence healthy food selection, cognitive loading, social modeling, decreasing tea spoon size	Limiting the consumption of daily sugar as the main risk factor of dental caries
		Tobacco and alcohol consumption	Nurchis et al. (34), Towner (35), Fakir and Bharati (36), Clarke et al. (37), Blaga et al. (38), Hempel-Bruder et al. (39), Cho et al. (40), Jenssen et al. (41), Drake et al. (42), Bhatt et al. (43)	Supportive interventions like as reminders, incentives, or access to resources such as quit lines or counseling services, graphic warning labels on cigarette packs, warning labels on alcohol bottles, highlighting the social norm of healthy behavior, making non-smoking the default option in public spaces, Increasing taxes on tobacco and alcohol products, Clinical Decision Support (CDS) intervention, timely feedback on tobacco or alcohol consumption	Limiting the consumption of alcohol and tobacco as main risk factors of oral diseases
	Medication adherence		Möllenkamp et al. (47), Kwan et al. (29), Luong et al. (49), Horne et al. (50), Rumi et al. (51), Ding et al. (48)	Reminders via SMS or text messages and motivational prompt messages, personalized nudge using machine learning	Increasing daily oral hygiene adherence
	Physical activity		Möllenkamp et al. (47), Kwan et al. (29), Landais et al. (54), Yang et al. (55), Forberger et al. (56, 57), Li et al. (58)	Reminders, planning prompts, feedback, behavioral contracts, salience nudges, gamification, wearable activity trackers	Increasing daily oral hygiene adherence
	Vaccination		Eze et al. (60), Levine et al. (64), Oyo-Ita et al. (65), Jacobson et al. (66), Sasaki et al. (67)	Patient reminders, education at village meetings, home, and facility-based health education, tailoring social norm	Receiving more oral disease preventive care such as fissure sealant and varnish fluoride
	Healthcare attendance		Möllenkamp et al. (47), Huf et al. (74), Friedman et al. (75), Boksmati et al. (76)	Financial incentives, reminders, planning prompts	Attending more dental checkup appointments
	Health insurance and retirement savings		Krishnan et al. (79), Marzilli Ericson (80), Beshears et al. (82), Dur et al. (83)	Framing, simplicity, social norms, defaults, sorting, callouts, labeling, reminders, personalized information. Framing the future time point around a fresh start date, social norm	Encouraging people to buy dental care insurance
Application of Nudge theory in oral health domains	Dental visit attendance		Wang et al. (21), Wang et al. (22)	Clarify the disadvantages of delaying treatment (gain and loss), social norm	Decreasing the missed dental appointments
	Oral health behavior change		Shariati et al. (20), Marciano et al. (23), White et al. (24)	Social norm, launch campaigns on the importance of good oral health, reminders, remind possible benefits of good oral hygiene routines, monetary incentive	Improving oral health behaviors such as tooth brushing

A unique characteristic of nudge interventions is their ability to influence behavior without being mandatory or restrictive. Instead of imposing choices on individuals, they use subtle design strategies to encourage them to make better decisions. In order to facilitate positive behaviors, these interventions utilize insights from behavioral science to shape the decision-making environment. The purpose of nudges is to gently guide individuals toward beneficial outcomes by making design choices that are effective and appealing.

This study has several limitations. Dated from the first major publishing of nudge theory in 2008 and limited to studies labeled as “nudges,” our search was limited to those that explicitly employ nudge theory in clinical practice enhancement. These criteria eliminate the probable number of clinical interventions before and after 2008 that employed the behavioral science theories on which “nudges” are based without using the term “nudge.” The criteria for English-language studies may have precluded relevant studies published in languages other than English.

## 4 Conclusion

The interventions made based on the nudge theory has been proven to be relatively efficient in conducting healthy decisions among patients. Despite the limited number of studies, their application in the field of oral health promotion has also yielded encouraging results. Besides, according to the common psychological mechanisms underlying many of the health behavioral patterns, reviewing the effective application of nudge theory in health domains rather than oral health might also be helpful in shaping new interventions in the field of oral health behavior changes. Therefore, we in this critical review, investigated the effectiveness of nudge strategies across domains including nutrition, tobacco and alcohol consumption, vaccination, medication adherence, visits to healthcare facilities, and health insurance purchase decisions. We argued that nudge theory could appropriately be applied to change the common risk factors of non-communicable diseases such as dental problems including sugar, tobacco and alcohol consumption. In addition, we presented that this theory might effectively enhance the recommended regular self-care behaviors such as oral hygiene practice among people. And finally, this theory might be a promising lead for encouraging people to buy appropriate insurance coverages including dental insurance. However, further exploration and clinical adaptation of these nudge interventions are highly recommended to enhance oral health promotion strategies holistically.

A comparative analysis of nudge interventions and non-nudge approaches is recommended for future research. The purpose of this exploration is to reveal the distinct impact of nudges on oral health behaviors, and thus provide valuable insights for advancing oral health interventions. A comparative analysis of nudge interventions and non-nudge approaches is recommended for future research. The purpose of this exploration is to reveal the distinct impact of nudges on oral health behaviors, and thus provide valuable insights for advancing oral health interventions.

## Data availability statement

The original contributions presented in the study are included in the article/supplementary material, further inquiries can be directed to the corresponding author.

## Author contributions

All authors listed have made a substantial, direct, and intellectual contribution to the work and approved it for publication.
